# Compensatory combination of romidepsin with gemcitabine and cisplatin to effectively and safely control urothelial carcinoma

**DOI:** 10.1038/s41416-020-0877-8

**Published:** 2020-05-11

**Authors:** Pawat Pattarawat, Tian Hong, Shelby Wallace, Yanchun Hu, Robert Donnell, Tzu-Hao Wang, Chia-Lung Tsai, Jinquan Wang, Hwa-Chain Robert Wang

**Affiliations:** 1 Department of Biomedical and Diagnostic Sciences, College of Veterinary Medicine, University of Tennessee, Knoxville, TN USA; 2 UT-ORNL Graduate School of Genome Science and Technology, University of Tennessee, Knoxville, TN USA; 3 Department of Biochemistry & Cellular and Molecular Biology, University of Tennessee, Knoxville, TN USA; 4 Genomic Medicine Research Core Laboratory, Chang Gung Memorial Hospital, Chang Gung University, Taoyuan, Taiwan; 5 Present Address: College of Veterinary Medicine, Sichuan Agricultural University, Chengdu, China; 6 Present Address: College of Veterinary Medicine, Xinjiang Agricultural University, Urumqi, Xinjiang China

**Keywords:** Cancer, Oncology

## Abstract

**Background:**

Human urothelial carcinoma (UC) has a high tendency to recur and progress to life-threatening advanced diseases. Advanced therapeutic regimens are needed to control UC development and recurrence.

**Methods:**

We pursued in vitro and in vivo studies to understand the ability of a triple combination of gemcitabine, romidepsin, and cisplatin (Gem+Rom+Cis) to modulate signalling pathways, cell death, drug resistance, and tumour development.

**Results:**

Our studies verified the ability of Gem+Rom+Cis to synergistically induce apoptotic cell death and reduce drug resistance in various UC cells. The ERK pathway and reactive oxygen species (ROS) played essential roles in mediating Gem+Rom+Cis-induced caspase activation, DNA oxidation and damage, glutathione reduction, and unfolded protein response. Gem+Rom+Cis preferentially induced death and reduced drug resistance in oncogenic H-Ras-expressing UC vs. counterpart cells that was associated with transcriptomic profiles related to ROS, cell death, and drug resistance. Our studies also verified the efficacy and safety of the Gem plus Rom+Cis regimen in controlling UC cell-derived xenograft tumour development and resistance.

**Conclusions:**

More than 80% of UCs are associated with aberrant Ras-ERK pathway. Thus the compensatory combination of Rom with Gem and Cis should be seriously considered as an advanced regimen for treating advanced UCs, especially Ras-ERK-activated UCs.

## Background

Urothelial carcinomas (UCs) account for >90% of urinary bladder cancer cases, which have been growing for the past 10 years in the US, reaching 80,470 new cases in 2019 and resulting in approximately 17,670 deaths.^1^ Conventional transurethral resection, chemotherapy, and Bacillus Calmette–Guerin immunotherapy are effective short-term treatments for UCs; however, >50% of UCs recur and progress to life threatening, advanced muscle-invasive UCs (MIUCs).^2–4^ Either a combination of gemcitabine (Gem) and cisplatin (Cis), the Gem plus Cis regimen, or a combination of methotrexate, vinblastine, adriamycin, and cisplatin (MVAC), are used as the standard treatment for advanced UCs.^5,6^ Because the Gem plus Cis regimen is less toxic than the MVAC regimen, the Gem plus Cis regimen is currently the preferred first-line chemotherapy for advanced UCs.^5,6^ Gem is a DNA synthesis-inhibiting agent, and Cis is a platinum-based DNA-damaging agent.^5,6^ However, despite the initial high response rates with these regimens, the overall 5-year survival rate of MIUC patients is <35% largely due to drug resistance and cancer recurrence.^2–4,7^ Gem resistance involves ribonucleotide reductase-catalysed DNA synthesis, activation of survival extracellular signal-regulated kinase (ERK) (Raf-Mek-Erk) pathway, unfolded protein responses (UPR), etc.^3–5,7,8^ Cis resistance involves the induction of glutathione (GSH)-dependent detoxification, poly(ADP-ribose) polymerase (PARP)-involved DNA repair, etc.^3,7,9^ Thus advanced regimens are urgently needed to effectively control advanced UC development and recurrence.^10,11^

Our research revealed that a combination of romidepsin (Rom, FK228) and Cis synergistically induces death and reduces drug resistance in UC cells via elevation of reactive oxygen species (ROS) to activate caspases and deplete GSH.^12^ Rom is a histone deacetylase inhibitor approved by the U.S. Food and Drug Administration (FDA) to treat T cell lymphoma.^13,14^ However, the therapeutic value of Rom for solid tumours is still unclear.^14,15^ Our studies also revealed that Rom is preferential to induce apoptosis and reduce drug resistance in oncogenic H-Ras-expressing cells vs. counterpart cells, via elevation of ROS and induction of the ERK pathway to activate caspases and deplete GSH.^16–20^ Although Cis alone is ineffective in inducing GSH depletion, Cis enhances the ability of Rom to deplete GSH.^12^ Accordingly, the integration of Rom into the standard Gem plus Cis regimen, resulting in Gem plus Rom+Cis, may advance therapeutics to effectively control UC development and recurrence.

In this communication, we demonstrated the ability of the triple combination Gem plus Rom+Cis regimen to synergistically induce death and reduce drug resistance in various UC cell lines in vitro. We investigated the mechanisms for Gem+Rom+Cis’s ability to control UC cells. We also demonstrated in vivo studies to verify the efficacy of Gem plus Rom+Cis regimen in controlling UC cell-derived xenograft (CDX) tumour development and resistance.

## Methods

### Cell cultures and reagents

Human UC J82, T24, SW780 (American Type Culture Collection [ATCC], Rockville, MD), and oncogenic H-Ras(V12)-expressing J82-Ras cells were maintained in Dulbecco’s modified Eagle’s medium with 5% heat-inactivated foetal bovine serum, 100 U/mL penicillin, and 100 µg/mL streptomycin.^16^ The J82-Ras cell line was established from the ectopic expression of the oncogenic H-Ras gene in J82 cells by constant transfection with pcDNA4/TO-E-H-ras plasmid.^16^ Cultures were maintained in 5% CO_2_ at 37 °C and sub-cultured every 2–3 days. Stock solutions of Rom, Cis, and Gem (Medkoo, Chapel Hill, NC, USA), U0126 (Cell Signaling, Beverly, MA), chloromethyl-dichlorodihydrofluorescein-diacetate (CM-H_2_DCF-DA) (Invitrogen, Carlsbad, CA, USA), and ML171 (EMD Millipore, Billerica, MA, USA) were prepared in dimethyl sulfoxide. Stock solution of *N*-acetyl-*L*-cysteine (NAC) (Alexis, San Diego, CA, USA) were prepared in distilled water. Stock solutions were diluted in culture medium for assays. Dosages of each reagent used in in vitro or in vivo assays are listed in Supplementary Tables S1 and S2, respectively.

J82 cells were constantly transfected with the pcDNA3.1^+^/human GRP78/BiP (binding immunoglobulin protein) plasmid DNA (GenScrpit, Piscataway, NJ, USA) and the psi-nU6.1/GRP78 short hairpin loop RNA (shRNA) plasmid DNA (GeneCopoeia, Rockville, MD), using TurboFect transfection reagent (Thermo Fisher, Waltham, MA, USA), to generate BiP-expressing J82 cell lines (J82-BiP-1 and J82-BiP-2) and BiP-downregulated J82 cell lines (J82-shBiP-1 and J82-shBiP-2) after selection with 1000 μg/mL of G418 (Corning, Corning, NY, USA) or 0.8 μg/mL of puromycin (Sigma-Aldrich, St. Louis, MO, USA), respectively. The targeted sequences for J82-shBiP-1 and J82-shBiP-2 were GCCTGACACCTGAAGAAATCG and GGAACCATCCCGTGGCATAAA, respectively.

### Cell viability

Cultured cells were treated with anticancer agents for 48 h. A methyl thiazolyl tetrazolium (MTT) assay kit (Travigen, Gaithersburg, MD, USA) was used to quantify cell viability with an enzyme-linked immunosorbent assay plate reader (Bio-Tek, Winooski, VT) at 570 nm.^12,16–21^ Relative values of cell viability in treated cultures were normalised by the value determined in untreated counterpart cells, set as 100%.

### Immunoblotting

Cells were treated for either 24 or 48 h, cell lysates were prepared, and protein concentrations were measured using the BCA assay (Thermo, Rockford, IL, USA).^12,16–21^ Equal amounts of cellular proteins were resolved by electrophoresis in 10% sodium dodecyl sulfate–polyacrylamide gels and transferred to nitrocellulose filters for immunoblotting, using specific antibodies to detect Ras, phosphorylated Erk1/2 (p-Erk1/2), Erk1/2, NADPH oxidase-1 (Nox-1), β-actin (Santa Cruz, Santa Cruz, CA, USA), phosphorylated Mek1/2 (p-Mek1/2), Mek1/2, BiP, and PARP (Cell Signaling). Concentrations of each antibody and blocking agent are shown in Supplementary Table S3. Antigen–antibody complexes on filters were detected by the SuperSignal West Dura Kit (Thermo). Levels of specific phosphorylation of Mek1/2 (p-Mek1/2) and Erk1/2 (p-Erk1/2) were calculated by normalising the levels of p-Mek1/2 and p-Erk1/2 with the levels of Mek1/2 and Erk1/2, respectively, then the level set in control cells as 1 (*X*, arbitrary unit). Levels of Ras, Nox-1, BiP, and PARP were calculated by normalising with the level of β-actin, and the level set in control cells as 1 (*X*, arbitrary unit).

### ROS measurement

Cells were treated with anticancer agents for 48 h. Cells were then labelled with 5 µmol/L CM-H_2_DCF-DA to measure ROS levels by flow cytometry using the Multicycle software (Phoenix, San Diego, CA, USA).^19–21^ The relative ROS level was measured and normalised by the level determined in untreated counterpart cells, set as 1 (*X*).

### Caspase activity assay

Cells were treated with anticancer agents for 24 h. Caspase-3/7 activity in cells was measured using a Caspase-Glo Assay Kit (Promega, Madison, WI, USA) with a luminometer plate reader (Bio-Tek).^12,17–21^ The relative caspase-3/7 activity was determined and normalised by cell viability, then the relative values were normalised by the value determined in untreated counterpart cells, set as 1 (*X*).

### Annexin-V apoptosis assay

Cells were treated with anticancer agents for 24 h. An annexin-V-fluorescein isothiocyanate apoptosis detection kit with propidium iodide (BD, San Jose, CA, USA) was used to measure the percentage of cells (%) undergoing apoptotic cell death by flow cytometry using the Multicycle software (Phoenix).^18,19^

### Clonogenic assay

Triplicates of 5 × 10^3^ cells were seeded in 60-mm culture dishes. Cultures were replaced with fresh medium after treatments and maintained for 7–14 days.^12,16,21^ Growing colonies (>30 cells) in untreated control cultures and drug-treated cultures were stained with crystal violet (0.5% w/v) on days 7 and 14, respectively. Cell colonies were counted and analysed using the TotalLab TL100 software (Newcastle, Tyne, UK, USA). Relative colony formation was determined and normalised by the value determined in untreated counterpart cells, set as 100%.

### GSH measurement

Cells were treated with anticancer agents for 48 h. Intracellular GSH levels were then measured with a QuantiChrom Glutathione Assay Kit (BioAssay, Hayward, CA, USA) using GSH disulfide as a standard.^21^ Relative GSH level was normalised by the value determined in untreated counterpart cells, set as 100%.

### DNA damage

DNA damage was detected with a comet assay,^22^ as preformed previously.^21^ In brief, after 24 h of treatment, 2 × 10^4^ cells/mL in phosphate-buffered saline (PBS) were mixed with an equal volume of 1% low-melting agarose (Fisher, Fair Lawn, NJ) and placed on agarose-coated slides. Slides were then lysed in an alkaline solution (1.2 M NaCl, 100 mM Na2EDTA, 0.26 M NaOH, TritonX 100, pH 13) overnight at 4 °C, electrophoresed in an alkaline buffer (0.3 M NaOH, 1 mM Na2-EDTA, pH >13) at 20 V/40 mA for 25 min, stained with propidium iodide, and examined with a fluorescence microscope (Carl Zeiss, Thornwood, NY). Fifty nuclei per slide were scored for tail moment (% DNA in tail × tail length) using the CometScore software (Tritek, Sumerduck, VA).^21^ The relative value of DNA damage was determined and normalised by the value determined in untreated counterpart cells, set as 1 (*X*).

### DNA oxidation

DNA oxidation was detected using a modified comet assay.^23^ Briefly, after 24 h of treatment, cells were seeded onto agarose-coated slides. Slides were immersed in lysis solution (2.5 M NaCl, 0.1 M Na_2_-EDTA, and 10 mM Tris, pH 10) overnight at 4 °C and then incubated with a reaction buffer (40 mM HEPES, 0.1 M KCl, 0.5 mM Na_2_-EDTA, and 0.2 mg/mL bovine serum albumin, pH 8) with and without formamidopyrimidine-DNA glycosylase (Fpg) for 30 min at 37 °C. Slides were then placed in an alkaline buffer (0.3 M NaOH, 1 mM Na_2_-EDTA, pH >10), electrophoresed, rinsed with a neutralisation buffer (0.4 M Tris, pH 7.5), stained with propidium iodide, examined with a fluorescence microscope, and analysed with the CometScore software (Tritek).^21,23^ The relative value of DNA oxidation was determined and normalised by the value determined in untreated counterpart cells, set as 1 (*X*).

### CDX model

Five-to-6-week old, female immuno-deficient athymic nu/nu (nude) mice (Envigo, Indianapolis, IN, USA) were used to establish CDX model. In brief, 2.5 × 10^6^ J82-Ras or T24 cells were mixed with Matrigel basement membrane matrix (BD) and inoculated subcutaneously into the flank areas of each nude mice to develop CDXs.^24^ Each cohort contained 4 mice calculated for power analysis (at a power of 80%) in order to detect a difference in tumour size of 80 ± 20 mm^3^ in this pilot study. Isoflurane (3–5%) (Zoetis, NJ, USA) was used as an anaesthesia by inhalation during inoculation. Mice were housed in sterile cages in a temperature-controlled room with 12-h light–dark cycle at the University of Tennessee Laboratory Animal Facility. Mice were provided with irradiated diet and water ad libitum. Animals were killed by CO_2_ exposure followed by cervical dislocation. The dosage and schedule of treatment are listed in Supplementary Table S2. All animal procedures were approved by the University of Tennessee Animal Care and Use Committee and were in accordance with the NIH Guide for the Care and Use of Laboratory Animals.

### In situ apoptosis detection

Paraffin-embedded tumour tissues were deparaffinised and rehydrated, followed by detection of apoptotic cells using the TACS 2 TdT-DAB In Situ Apoptosis Detection Kit (Trevigen, MD, USA). Cultures of J82-Ras cells were fixed with 3.7% formaldehyde and examined with the same kit to detect apoptotic cells. Samples were counterstained by methyl green.

### Histological examination

Tumour tissues were isolated, fixed in neutral-buffered formalin, and embedded in paraffin, followed by haematoxylin and eosin staining of tissue sections for histopathological examination.

### Transcriptomic analysis

Cellular RNAs were isolated with the Quick-RNA™ MicroPrep Kit (Zymo, Irvine, CA, USA) for transcriptomic analysis. The quality of RNAs was measured by an Agilent bioanalyser 2100 (Agilent, Santa Clara, CA, USA), and RNA samples with an RNA integrity number >7 were qualified for transcriptomic analysis. The Affymetrix GeneChip Human Transcriptome Array 2.0A (>245,000 coding and >40,000 non-coding transcripts) and the Affymetrix GeneChip® Scanner 3000 7G (ThermoFisher, Waltham, MA, USA) were used to detect gene expression levels. Data were analysed using Python scripts to identify genes whose expression was either significantly (false discovery rate (FDR) < 0.05) increased or decreased by >2-fold. Custom gene ontology (GO)^25^ was used to analyse the functional enrichment of the modulated genes.

### Statistical analysis

Student *t* test was used to analyse statistical significance. *p* values were adjusted for multiple comparisons using the Simes method^26^ with the Stata 16 software (StataCorp LLC, College Station, TX, USA). Statistical significance was indicated by *p < 0.05, **p < 0.01, ***p < 0.001; a *p* value <0.05 was considered significant. Combination index analysis was performed using the method by Chou and Talalay^27^ via the CompuSyn software suite (Paramus, NJ, USA). Combination indices <1, =1, and >1 indicate synergistic, additive, and antagonistic effects, respectively.

## Results

### ROS- and ERK-Nox-dependent cell death synergistically induced by combined Rom, Cis, and Gem

**Fig. 1:**
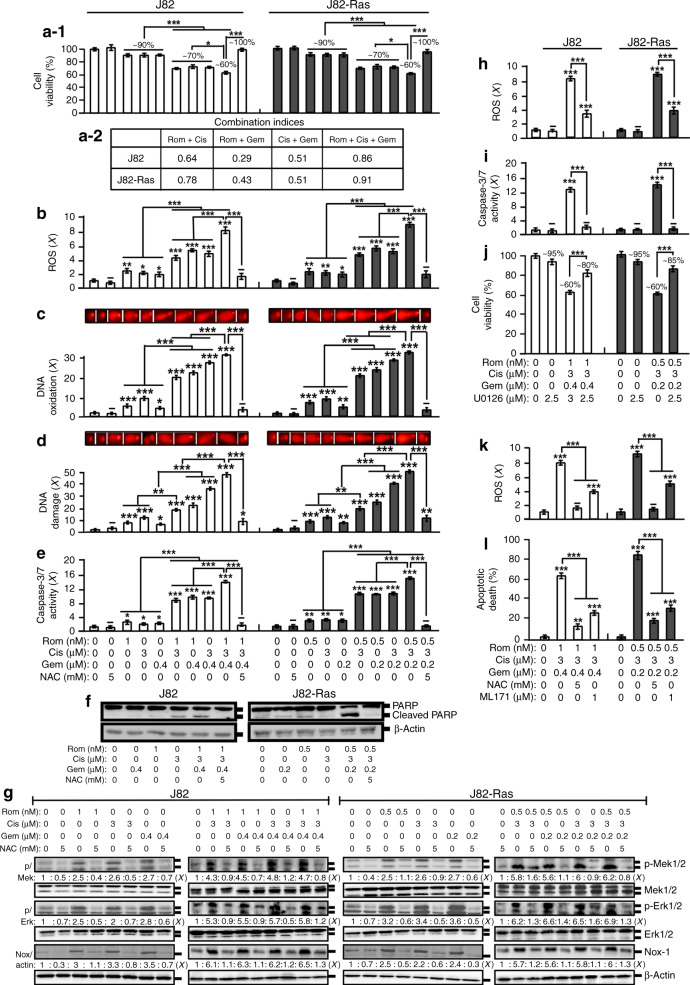
ROS- and ERK-Nox-dependent cell death synergistically induced by combined Rom, Cis, and Gem. **a**–**g** J82 and J82-Ras cells were treated with Rom, Cis, and/or Gem at their IC_10_ doses in the presence and absence of NAC for 48 h (**a**, **f**) or 24 h (**b**–**e**, **g**). **h**–**j** Cells were treated with Rom, Cis, and/or Gem in the presence and absence of U0126 for 24 h (**h**, **i**) or 48 h (**j**). **k**, **l** Cells were treated with Rom+Cis+Gem in the absence and presence of NAC or ML171 for 24 h (**k**) or 48 h (**l**). **a-1**, **j** Cell viability was measured with an MTT assay kit, and relative cell viability was normalised by the value determined in untreated counterpart cells, set as 100%. **a-2** Combined effects (**a-1**) were evaluated to reveal combination indices <1 for synergistic effects. **b**, **h**, **k** Relative ROS levels were measured and normalised by the level determined in untreated counterpart cells, set as 1 (*X*, arbitrary unit). **c** DNA oxidation was measured by an Fpg-modified comet assay and normalised by the value of average tail moment determined in untreated control cells, set as 1 (*X*, arbitrary unit). **d** DNA damage was measured by an alkaline comet assay and normalised by the value of average tail moment determined in untreated control cells, set as 1 (*X*, arbitrary unit). Representative images of DNA oxidation and damage (**c**, **d**) are shown. **e**, **i** Relative caspase-3/7 activity was determined and normalised by cell viability, and then the relative values were normalised by the value determined in untreated counterpart cells, set as 1 (*X*, arbitrary unit). **f**, **g** Cell lysates were prepared and analysed by immunoblotting using specific antibodies to detect the levels of PARP, cleaved PARP, p-Mek1/2, Mek1/2, p-Erk1/2, Erk1/2, and Nox-1, with β-actin as a control, and these levels were quantified by densitometry. Levels of specific phosphorylation of Mek1/2 (p/Mek) and Erk1/2 (p/Erk) were calculated by normalising the levels of p-Mek1/2 and p-Erk1/2 with the levels of Mek1/2 and Erk1/2, respectively, then the level set in control cells as 1 (*X*, arbitrary unit). Levels of Nox-1 (Nox/actin) were calculated by normalising with the level of β-actin and the level set in control cells as 1 (*X*, arbitrary unit). **l** Apoptotic cell population (%) was measured by flow cytometry with an annexin-V-FITC apoptosis detection kit. Columns, mean of triplicates; bars, SD. *p* Value was adjusted for multiple comparisons by using the Simes method. Statistical significance is indicated by *p < 0.05, **p < 0.01, ***p < 0.001. All results are representative of three independent experiments.

**Fig. 2:**
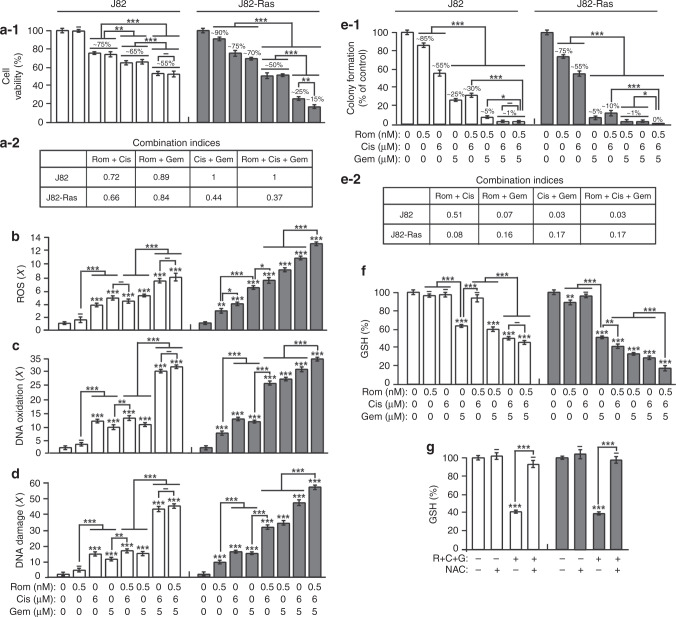
Preferential induction of cell death and suppression of drug resistance by Rom+Cis+Gem in J82-Ras vs. J82 cells. J82 and J82-Ras cells were treated with Rom, Cis, and/or Gem in the presence or absence NAC. **a-1** Cell viability was measured with an MTT assay kit, and relative cell viability was normalised by the value determined in untreated counterpart cells, set as 100%. **a-2** Combined effects (**a-1**) were determined to reveal combination indices <1 or =1 for synergistic or additive effects, respectively. **b** Relative ROS levels were measured and normalised by the level determined in untreated counterpart cells, set as 1 (*X*, arbitrary unit). **c** DNA oxidation was measured by an Fpg-modified comet assay and normalised by the value of average tail moment determined in untreated control cells, set as 1 (*X*, arbitrary unit). **d** DNA damage was measured by an alkaline comet assay and normalised by the value of average tail moment determined in untreated control cells, set as 1 (*X*, arbitrary unit). **e-1** Clonogenic survival was measured by a clonogenic assay. Relative colony formation was normalised by the value determined in untreated counterpart cells, set as 100%. **e-2** Combined effects (**e-1**) were determined. **f**, **g** GSH content was determined, and relative GSH level was normalised by the value determined in untreated counterpart cells, set as 100%. Columns, mean of triplicates; bars, SD. *p* Value was adjusted for multiple comparisons by using the Simes method. Statistical significance is indicated by *p < 0.05, **p < 0.01, ***p < 0.001. All results are representative of three independent experiments.

To investigate the ability of combined Gem with Rom and Cis in controlling UC cells, we initially determined their inhibitory concentrations (ICs) for the UC J82 and the oncogenic H-Ras-expressing J82-Ras cells. Subsequently, we investigated the ability of combined agents at their cognate IC_10_ doses (Supplementary Table S1) to reduce J82 and J82-Ras cell viability (Fig. 1a-1). Using the Chou–Talalay method,^27^ we determined that double and triple combinations of Rom, Cis, and Gem synergistically reduced viability of J82 and J82-Ras cells (Fig. 1a-2), indicating that a combination of these agents was able to synergistically induce UC cell death.

ROS elevation plays an important role in the cytotoxicity of Rom, Cis, and Gem.^28–32^ Studying the ROS content in cells, we detected that treatment with these agents individually at their cognate IC_10_ doses resulted in ROS elevation (Rom>Cis>Gem) (Fig. 1b). ROS were increasingly induced by triple>double>single agents. ROS elevation has been shown to correlate with oxidative lesions in DNA, DNA damage, and cell death.^33^ We detected that DNA oxidation was induced by Cis>Rom>Gem and induced by triple>double>single agents in J82 and J82-Ras cells (Fig. 1c). Levels of DNA damage were closely correlated with levels of DNA oxidation (Fig. 1d). Rom and Cis appeared to be more effective than Gem in inducing DNA oxidation and DNA damage. Studying apoptosis-related pathways revealed that caspase-3/7 activation was induced by Rom but not Cis and Gem at their IC_10_ doses in J82 cells. In contrast, caspase-3/7 was induced by Rom, Cis, and Gem in J82-Ras cells, and caspase-3/7 was induced by triple>double>single agents (Fig. 1e). The triple combination induced cleavage of PARP to a higher degree than the single agent (Fig. 1f). Using NAC to block ROS completely abrogated cell death, DNA oxidation, DNA damage, caspase-3/7 activation, and PARP cleavage induced by Rom+Cis+Gem (Fig. 1a–f), indicating that Rom+Cis+Gem induced cell death, DNA oxidation, DNA damage, caspase-3/7 activation, and PARP cleavage in an ROS-dependent manner.

We showed that the ERK-Nox pathway is involved in ROS elevation and cell death induced by Rom.^19^ We also detected that double and triple combinations induced higher levels of phosphorylated Mek1/2 and Erk1/2, as well as Nox-1, than single agents, indicating that double and triple combinations induced higher activation of the ERK-Nox pathway than single agents (Fig. 1g and Supplementary Figs. S1 and S2b). Co-treatment with NAC abrogated the activation of the ERK-Nox pathway by Rom, Cis, and Gem, indicating that ROS elevation also played a role in ERK-Nox pathway activation induced by Rom, Cis, and Gem.

To verify the role of the ERK-Nox pathway in cell death induced by Rom+Cis+Gem, we used the Mek1/2 inhibitor U0126 to block the ERK pathway and the specific inhibitor ML171 to suppress the Nox-1 activity in cells. Blockage of the ERK pathway resulted in suppressing Rom+Cis+Gem-induced ROS elevation, caspase-3/7 activation, and cell death (Fig. 1h–j). Inhibition of Nox-1 by ML171 or overall ROS by NAC resulted in significant reduction of Rom+Cis+Gem-induced ROS and apoptotic cell death (Fig. 1k, l). The results indicated an important role the ERK-Nox pathway played in the induction of ROS elevation, caspase activation, and apoptotic cell death induced by Rom+Cis+Gem.

### Preferential induction of cell death and suppression of drug resistance by Rom+Cis+Gem in J82-Ras vs. J82 cells

We showed that Rom preferentially induces death in J82-Ras vs. J82 cells.^16–20^ To reveal the ability of Rom+Cis+Gem to control J82-Ras vs. J82 cells, we combined 0.5 nmol/L Rom (the IC_10_ dose for J82-Ras but non-cytotoxic to J82), 6 µmol/L Cis (the IC_25_ dose for both J82 and J82-Ras), and 5 µmol/L Gem (IC_25_ dose for J82 but >IC_25_ for J82-Ras). Double or triple combinations resulted in preferentially reducing viability of J82-Ras (ranging from ~50 to ~15%) vs. J82 (~65 to ~55%) cells, and the triple combination reduced viability of J82-Ras to ~15% vs. J82 to ~55% (Fig. 2a-1). Combination indices indicated that all the combinations synergistically induced J82-Ras cell death. Rom+Cis and Rom+Gem synergistically induced J82 cell death, but Cis+Gem and Rom+Cis+Gem additively induced J82 cell death (Fig. 2a-2). Also, Rom+Cis+Gem was more effective than single agents and double combinations in preferential induction of ROS elevation, DNA oxidation, and DNA damage in J82-Ras vs. J82 cells (Fig. 2b–d). These results indicated that Rom+Cis+Gem was effective and synergistic in preferentially inducing cell death, ROS elevation, DNA oxidation, and DNA damage in J82-Ras vs. J82 cells.

Clonogenic survival rate serves as an index for drug resistance of cancer cells.^34^ Drug resistance to Cis for survival is associated with GSH-based detoxification.^35,36^ GSH is an ROS scavenger, and depletion of GSH increases cellular susceptibility to ROS-induced apoptosis.^37^ We showed that, although Cis by itself fails to reduce GSH, Cis enhances the ability of Rom to reduce GSH in various cancer cells.^12,21^ Studying drug resistance, we detected that Rom and Gem, but not Cis, preferentially reduced clonogenic survival in J82-Ras vs. J82 cells (Fig. 2e-1). Rom+Cis and Rom+Gem reduced clonogenic survival of J82-Ras to ~10% and ~1% vs. J82 cells to ~30% and ~5%, respectively, indicating that the two double combinations preferentially reduced clonogenic survival of J82-Ras vs. J82 cells. However, Cis+Gem reduced clonogenic survival of J82-Ras and J82 cells at a similar level (~1%). Rom+Cis+Gem was able to completely suppress J82-Ras clonogenic survival to 0%, while minor survival (~1%) occurred in J82 cells. Combination indices indicated that all the combinations synergistically reduced clonogenic survival in J82 and J82-Ras cells (Fig. 2e-2). Accordingly, Rom+Cis+Gem was more effective than single agents and double combinations to preferentially reduce drug resistance in J82-Ras vs. J82 cells. Rom+Cis+Gem was also more effective than single agents and double combinations in the preferential reduction of GSH in J82-Ras vs. J82 cells (Fig. 2f). Treatment with Rom or Gem, but not Cis, resulted in significantly reducing GSH; and Gem appeared to reduce GSH to lower levels than Rom in both J82 and J82-Ras cells. Although treatment with Cis reduced clonogenic survival (Fig. 2e-1), it did not reduce GSH in either cells (Fig. 2f). Interestingly, combining Rom with Cis resulted in profoundly suppressing clonogenic survival in J82 cells (Fig. 2e-1), but it did not reduce GSH (Fig. 2f). Blockage of ROS with NAC effectively abrogated Rom+Cis+Gem-reduced GSH (Fig. 2g). These results indicated that GSH depletion was involved in Rom+Cis+Gem-induced suppression of drug resistance in J82 and J82-Ras cells in an ROS-dependent manner. Overall, ROS elevation, DNA oxidation, DNA damage, and GSH depletion were relatively, but not fully, correlated with induced cell death and reduced clonogenic survival by these agents, indicating that other mechanisms involved in the reduction of cell viability and drug resistance by Rom, Cis, and Gem remain to be determined.

### Rom+Cis+Gem synergistically induced death and suppressed drug resistance in SW780 cells

Clarifying whether the ability of the triple combination Rom+Cis+Gem to synergistically induce death and reduce drug resistance was unlimited to the MIUC J82 and J82-Ras cells, we included the transitional cell carcinoma SW780 cell line in our studies. We determined IC doses of Rom, Cis, and Gem for SW780 cells. We detected that Rom+Cis+Gem was more effective than single and double agents to induce cell death, and all the combinations synergistically induced death in SW780 cells (Supplementary Fig. S3a-1, a-2). Similar to J82 and J82-Ras cells, treatment with Rom, Cis, and/or Gem induced the ERK-Nox pathway, ROS elevation, caspase-3/7 activation, DNA oxidation, and DNA damage (Supplementary Fig. S3b–f) that were relatively increased by triple>double>single agents. The inhibition of ROS with NAC blocked Rom+Cis+Gem-induced ERK-Nox pathway, caspase-3/7 activation, DNA oxidation, and DNA damage. The inhibition of the ERK pathway with U0126 blocked Rom+Cis+Gem-induced p-Erk1/2, Nox-1, ROS, and caspase-3/7 (Supplementary Fig. S3b–d). NAC blockage of ROS or ML171 inhibition of Nox-1 resulted in suppressing Rom+Cis+Gem-induced ROS and apoptotic cell death (Supplementary Fig. S3g, h). These results indicated that ROS elevation was essential for Rom+Cis+Gem-induced DNA oxidation and damage, and the ERK-Nox pathway and ROS were mutually reliant and were both required for caspase-3/7 activation and apoptotic death induced by Rom+Cis+Gem.

We also detected the ability of Rom+Cis+Gem, at their cognate IC_10_ doses, to completely suppress clonogenic survival that was more effective than single and double agents (Supplementary Fig. S3i). GSH content was reduced by triple>double>single agents, and NAC inhibition of ROS blocked Rom+Cis+Gem-induced GSH depletion (Supplementary Fig. S3j), indicating an essential role of ROS elevation for GSH depletion. Although Cis was ineffective to induce GSH depletion (Supplementary Fig. S3j), it enhanced the ability of Rom and Gem to suppress clonogenic survival and deplete GSH (Supplementary Fig. S3i, j). The results in SW780 cells were consistent with results from J82 and J82-Ras cells, indicating that the ability of Rom+Cis+Gem to synergistically induce cell death and reduce drug resistance was not limited to one type of UC cells.

### Transcriptomic profiles associated with Rom+Cis+Gem

**Fig. 3:**
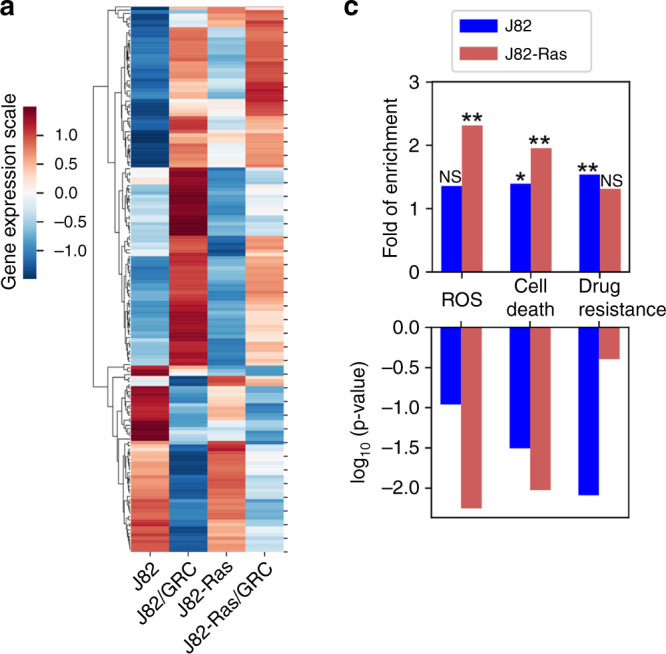

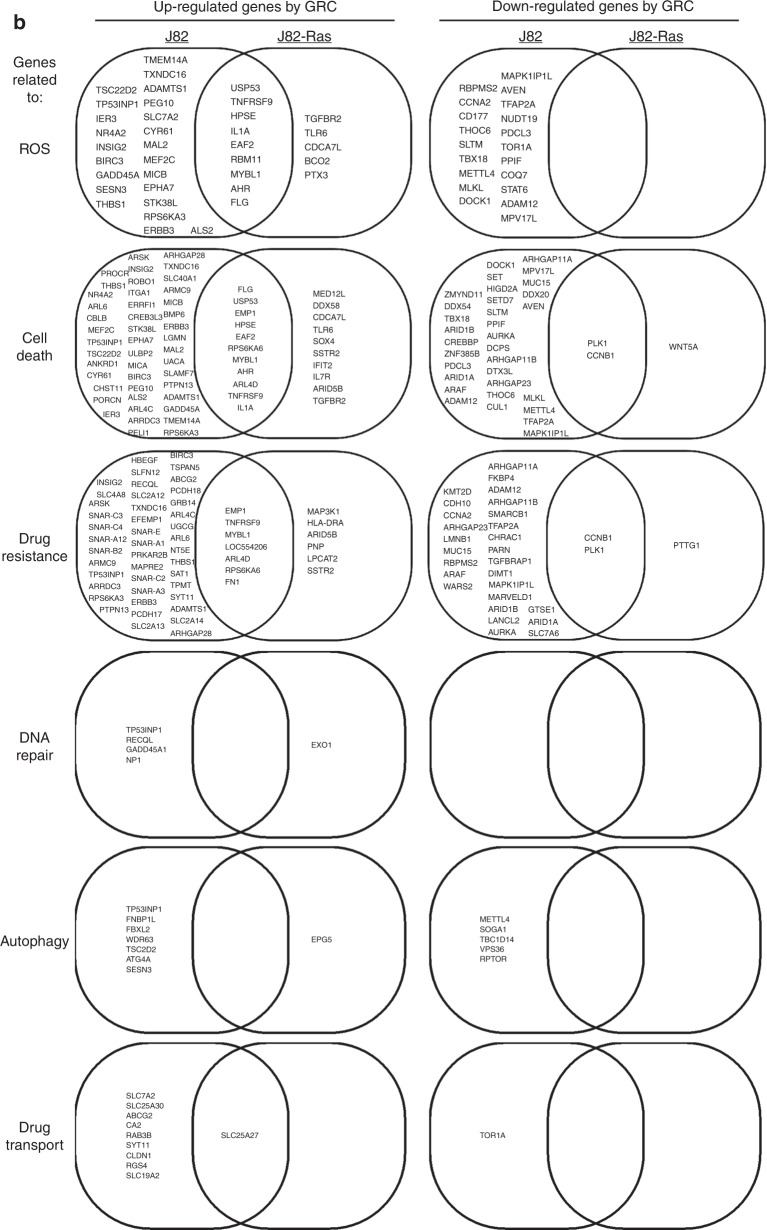
Transcriptomic profiles associated with Rom+Cis+Gem. J82 and J82-Ras cells were treated with Rom+Cis+Gem at their IC_10_ doses for J82-Ras cells for 48 h to induce differential cell death. **a** Heatmap of 179 differentially expressed genes (>2-fold change, FDR < 0.05), induced by Rom+Cis+Gem, that are functionally associated with ROS, cell death, or drug resistance. Colour code represents the *z*-score of the expression. **b** Venn diagram of differentially regulated genes, induced by Rom+Cis+Gem, functionally associated with ROS, cell death, drug resistance, DNA repair, autophagy, or drug transport in J82 and J82-Ras cells were compared. **c** Quantification of upregulated ROS-, cell death-, and drug resistance-associated gene expression levels. Upper panel shows the folds of increases. Lower panel shows the associated *p* values. Fisher’s exact test was used to obtain statistical significance: *p < 0.05, **p < 0.01, not significant (NS) *p* > 0.05.

To detect molecular changes associated with the ability of Rom+Cis+Gem to modulate ROS, cell death, and drug resistance, we used the Affymetrix GeneChip Human Transcriptome Array 2.0A, which carries >245,000 coding and 40,000 non-coding transcripts, to detect gene expression levels in cells. Initially, >25,000 coding transcripts were detected in J82-Ras and J82 cells. We quantified functional enrichment through GO analysis,^25^ which is based on two lists of 2087 and 3720 annotated genes associated with ROS and cell death, respectively. Because “drug resistance” is not a standard ontology term, we used a gene set from the database of Genomic Elements Associated with drug Resistance,^38^ which provides a list of 2895 drug resistance-associated genes. Our transcriptome analysis revealed 297 and 181 genes upregulated and downregulated (>2-folds, FDR < 0.05), respectively, by Rom+Cis+Gem in J82 cells; and 88 and 15 genes were upregulated and downregulated, respectively, in J82-Ras cells (Fig. 3a). In J82-Ras cells, 13 ROS-associated and 22 cell death-associated genes were significantly upregulated by Rom+Cis+Gem, giving rise to significant enrichment of these two functions (Fig. 3b, *p* < 0.11, Fisher’s exact test). However, upregulated drug-resistant genes in Rom+Cis+Gem-treated J82-Ras cells were not significantly enriched (*p* = 0.40, Fisher’s exact test). In contrast, 50 drug resistance-associated genes were upregulated in Rom+Cis+Gem-treated J82 cells, giving rise to significant enrichment of this function (*p* = 0.008, Fisher’s exact test). Although upregulation of 32 ROS-associated and 57 cell death-associated genes was induced by Rom+Cis+Gem in J82 cells, ROS-associated genes were not significantly enriched (*p* = 0.11, Fisher’s exact test). We also detected that, although Rom+Cis+Gem induced some of the commonly regulated genes associated with ROS, cell death, and drug resistance in both cells, much fewer genes were significantly induced in J82-Ras cells than in J82 cells (Fig. 3a, c). In addition, more genes associated with DNA repair, autophagy, and drug transport were upregulated by Rom+Cis+Gem in J82 than in J82-Ras cells. None of the DNA repair-associated genes was detectably downregulated in J82 and J82-Ras cells, and none of the autophagy- or drug transport-associated genes was detectably downregulated in J82-Ras cells, while a few of genes were downregulated in J82 cells (Fig. 3b). Taking these results together, our transcriptome analysis revealed that drug resistance-associated genes were significantly induced in J82 but not in J82-Ras cells, whereas the ROS-associated genes were significantly induced in J82-Ras but not in J82 cells. The higher level of cell death-associated genes significantly induced by Rom+Cis+Gem in J82-Ras than in J82 cells was closely correlated with the preferential induction of death in J82-Ras vs. J82 cells. The higher numbers of DNA repair-, autophagy-, and drug transport-associated genes induced by Rom+Cis+Gem in J82 than in J82-Ras cells were correlated with the preferential suppression of clonogenic survival in J82-Ras vs. J82 cells.

### BiP contributed to cell death induced by Rom+Cis+Gem

**Fig. 4:**
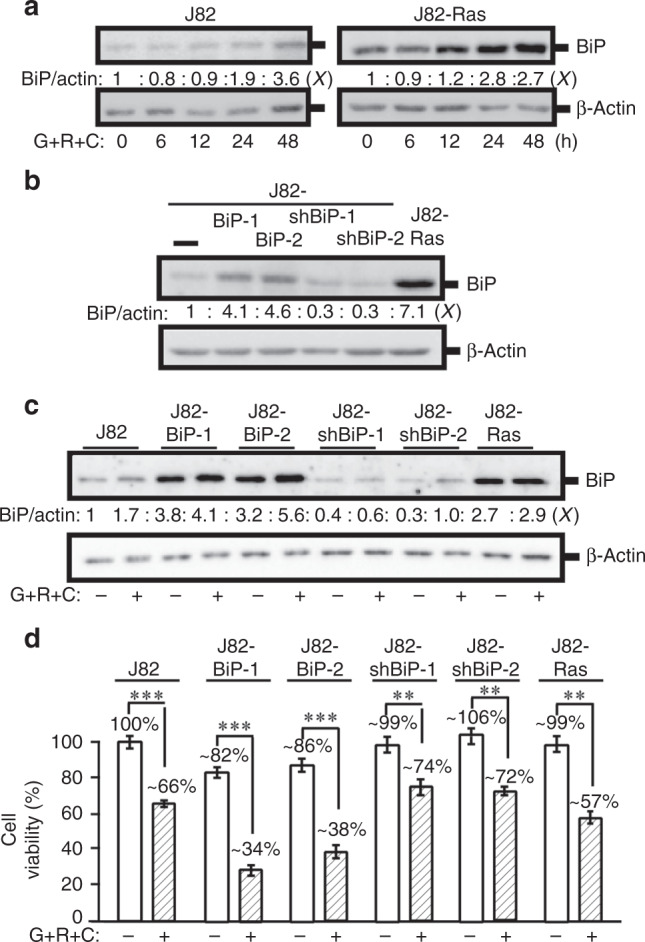
BiP contributed to cell death induced by Rom+Cis+Gem. **a** J82 and J82-Ras cells were treated with Rom+Cis+Gem at their IC_10_ doses for 0, 6, 12, 24, and 48 h. **b** J82 cells were constantly transfected with a BiP expression vector to result in J82-BiP-1 and -2 cell lines. J82 cells were constantly transfected with BiP-specific shRNA vectors to result in J82-shBiP-1 and -2 cell lines. **c**, **d** J82-BiP-1, BiP-2, shBiP-1, and shBiP-2, as well as J82-Ras cells were treated Rom+Cis+Gem. **a**–**c** Cell lysates were prepared and analysed by immunoblotting using specific antibodies to detect the levels of BiP with β-actin as a control, and these levels were quantified by densitometry. Levels of BiP (BiP/actin) were calculated by normalising with the level of β-actin, and the level set in control cells as 1 (*X*, arbitrary unit). **d** Cell viability was determined, and relative cell viability was normalised by the value determined in untreated counterpart cells, set as 100%. Columns, mean of triplicates; bars, SD. *p* Value was adjusted for multiple comparisons by using the Simes method. Statistical significance is indicated by **p < 0.01, ***p < 0.001. All results are representative of three independent experiments.

The BiP is known to support tumorigenesis and anti-apoptosis and is the key modulator for UPR involved in cellular response to stress, autophagy, and apoptosis.^39,40^ BiP helps the resistance to Gem,^8^ indicating an association between UPR with drug resistance. Our study detected that BiP was elevated by Rom+Cis+Gem in both J82 and J82-Ras cells (Fig. 4a), as well as in T24 and SW780 cells (Supplementary Fig. S4). Using gain- and loss-of-function approaches, we ectopically expressed BiP by constant transfection and knocked down BiP by specific shRNAs in J82 cells, resulting in J82-BiP-1 and -2, as well as shBiP-1 and -2 cell lines, respectively (Fig. 4b). Treatment with Rom+Cis+Gem induced increases of BiP in these cells (Fig. 4c). Interestingly, ectopic expression, but not knockdown, of BiP resulted in increased susceptibility of J82 cells to Rom+Cis+Gem for cell death; in contrast, knockdown of BiP appeared to increase moderate resistance to Rom+Cis+Gem (Fig. 4d). These results indicated a novel role of elevated BiP played in supporting cell death but not drug resistance in UC cells in response to Rom+Cis+Gem.

### Efficacy of the Gem plus Rom+Cis regimen in controlling J82-Ras CDXs

**Fig. 5:**
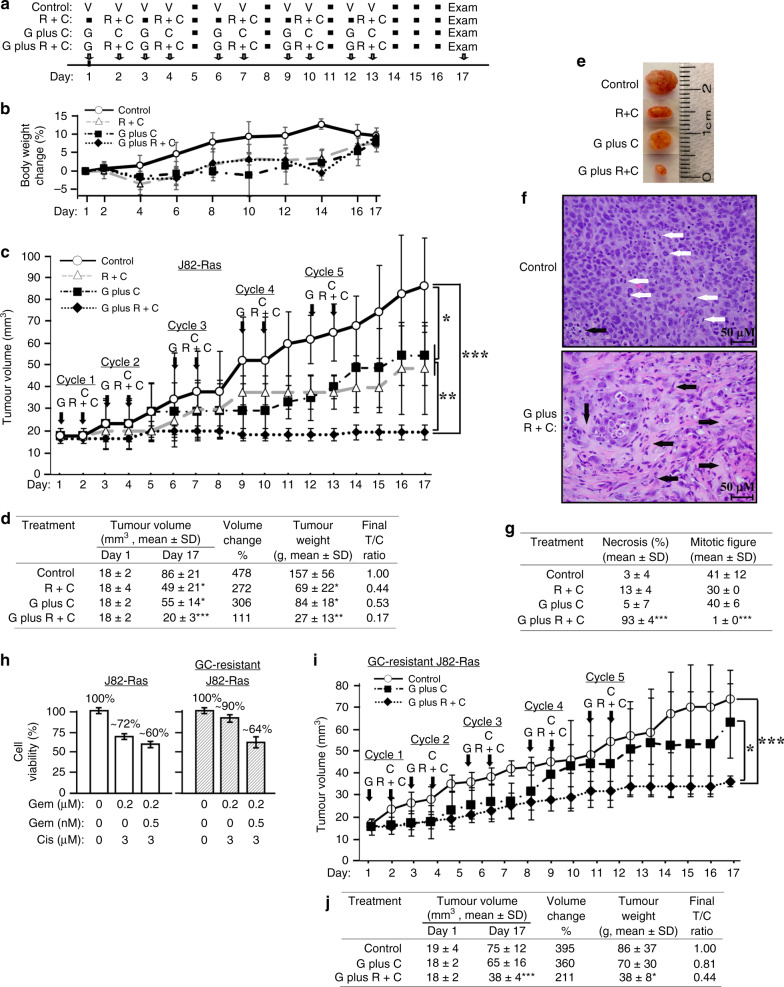
Efficacy of the Gem plus Rom+Cis regimen in controlling J82-Ras CDXs. **a** The immuno-deficient nu/nu (nude) mice, 4 per group, were injected (i.p.) with PBS (V, control), 1 mg/kg Rom mixed with 5 mg/kg Cis (Rom+Cis, R+C), 20 mg/kg Gem followed by 5 mg/kg Cis (Gem plus Cis, G plus C) and Gem plus Rom+Cis (G plus R+C) for 5 treatment cycles at the indicated days as scheduled with 0, 1, or 2 days of interval. **b** Body weight was measured every 2 days to determine body weight loss for revealing adverse side effects of a regimen on animals. **c** In all, 2.5 × 10^6^ J82-Ras cells were mixed with Matrigel and inoculated into the flank areas of nude mice. Tumour volume was measured with a calliper and determined with the formula (length × width^2^ × ½).^43^ Mice developing CDXs reaching ~18 mm^3^ were entered into the treatment study (day 1). Nude mice, four per group, were administered (i.p.) PBS (V, control), Rom+Cis (R+C), Gem plus Cis (G plus C), and Gem plus Rom+Cis (G plus R+C) at the indicated days for five treatment cycles. Tumour volume was measured daily. Mice were histopathologically examined during necropsy at day 17. **d** Tumour volume at days 1 and 17 is presented as mean ± SD. Changes of tumour volume (%) were calculated by T17 (tumour volume determined at day 17)/T1 (tumour volume determined at day 1). Average weight of tumours isolated at day 17 was measured as mean ± SD. Final tumour/control ratio (T/C) was calculated by T (mean tumour weight of treatment group)/C (mean tumour weight of control group) of tumours isolated from mice at day 17. **e** Representative tumours are shown. **f** Histological features of the representative tumours isolated from control mice and mice treated with Gem plus Rom+Cis are shown. White arrows indicate mitotic cells, and black arrows indicate necrosis area (irreversible damage). Images were taken at ×400; scale bar, 50 µm. **g** Necrosis areas were analysed using the ImageJ software,^62^ and mitotic cells were determined. Necrosis areas (%) and mitotic figures/cells, identified in tumours isolated from control mice and mice treated with combination regimens, were averaged from 10 high-power fields (HPFs) (×400). **h** GC-resistant J82-Ras cell line was established from growing tumours from mice treated with the Gem plus Cis regimen. Parental J82-Ras and GC-resistant J82-Ras cells were treated with Gem+Rom+Cis for 48 h, and cell viability was determined. **i** As performed above in **c**, 2.5 × 10^6^ GC-resistant J82-Ras cells were mixed with Matrigel and inoculated into nude mice to develop detectable CDXs, followed by treatments with the Gem plus Cis+Rom or the Gem plus Cis regimen. **j** The efficacy in controlling GC-resistant J82-Ras CDXs was determined as performed above in **d**. Statistical significance is indicated by *p < 0.05, **p < 0.01, ***p < 0.001.

In the standard protocol of the Gem plus Cis regimen to treat UC patients, 1000 mg/m^2^ Gem is given at days 1, 8, and 15, and 70 mg/m^2^ Cis is administered at day 2 (Supplementary Table S4).^5,6^ In treating lymphoma or refractory solid tumours, 8–17.5 mg/m^2^ Rom is given at days 1, 8, and 15 (Supplementary Table S2).^30,41^ Calculations based on NCI’s Equivalent Surface Area Dosage Conversion Factors^42^ suggest that administering 1000 mg/m^2^ Gem, 15 mg/m^2^ Rom, and 70 mg/m^2^ Cis to humans is equivalent to administering 324 mg/kg Gem, 5 mg/kg Rom, and 23 mg/kg Cis to mice (Supplementary Table S2). Considering the synergy and toxicity^5,41^ of Rom+Cis+Gem, we formulated dose-reduced combination regimens containing 20 mg/kg Gem, 1 mg/kg Rom, and/or 5 mg/kg Cis. As shown in Fig. 5a, b, using animal body weight loss to detect adverse side effects,^43^ we determined drug-administering schedules for tolerable regimens and protocols. Although mice administered with combination regimens appeared to gain weight less efficient than the control group, animals did not lose body weight (Fig. 5b) or show any visible adverse side effects, such as inability to move, eat, drink, etc. Administering mice (intraperitoneally) with the triple combination Gem plus Rom+Cis regimen for 2 consecutive cycles, followed by 1 day of intervals after the second, third, and fourth cycles of treatment (Supplementary Table S4) was well tolerated and safe to mice.

Determining the ability of the Gem plus Rom+Cis regimen to control tumour development in vivo, we implanted J82-Ras cells subcutaneously into the flank area of nude mice. Mice developing CDX tumours reaching ~18 mm^3^ were admitted into the treatment study. Tumour volume was measured, and histopathological examination was performed during necropsy (day 17) 4 days after the last treatment (day 13) (Fig. 5a, c). We observed that two treatment cycles of these regimens effectively controlled CDXs; however, tumours appeared to become resistant to the Rom+Cis and the Gem plus Cis regimens afterward (Fig. 5c). In contrast, Gem plus Rom+Cis was highly efficacious in controlling CDX development throughout treatment cycles. Analysis of final tumour volume revealed a growth of CDXs in 17 days to ~480%, ~270%, ~300%, and ~110% of their original volume in mice treated with vehicle, Rom+Cis, Gem plus Cis, and Gem plus Rom+Cis, respectively (Fig. 5d). Comparing tumour weights showed that the final tumour/control ratio (T/C) of tumours isolated at day 17 from mice treated with Rom+Cis, Gem plus Cis, and Gem plus Rom+Cis were at 0.44/1.0, 0.53/1.0, and 0.17/1.0, respectively. The result verified the efficacy of these regimens in controlling CDX development. The results also indicated that the Gem plus Rom+Cis regimen was more efficacious than double combination regimens in controlling CDX development and resistance.

Histological examination of isolated tumours (Fig. 5e) revealed an average of ~41, ~30, ~40, and ~1 mitotic figures per high-power field (HPF) in tumour tissues isolated from mice treated with vehicle, Rom+Cis, Gem plus Cis, and Gem plus Rom+Cis, respectively (Fig. 5f, g). Necrosis area was detectably higher in tumours isolated from mice treated with Gem plus Rom+Cis than in mice treated with vehicle, Rom+Cis, or Gem plus Cis at an average of ~93% vs. ~3% vs. ~13% vs. ~5%, respectively. We also used the terminal deoxynucleotidyl transferase dUTP nick end labelling (TUNEL) assay to detect apoptotic cells in tumour tissues and cultured cells. The TUNEL assay determined that Gem+Rom+Cis induced apoptosis of cultured cells in vitro but did not increase the apoptotic cell population in tumours (Supplementary Fig. S5). These results indicated that the Gem plus Rom+Cis regimen inhibited proliferation and induced death via necrosis but not via apoptosis of tumour cells more efficaciously than the Rom+Cis and the Gem plus Cis regimen in treated animals.

To determine the ability of Gem plus Rom+Cis regimen to control resistant tumours from animals treated with the standard Gem plus Cis regimen, we isolated growing J82-Ras CDXs from animals after five cycles of treatment with the Gem plus Cis regimen (Fig. 5c) and developed into GC-resistant J82-Ras cells. GC-resistant J82-Ras cells were highly resistant to Gem+Cis treatment with an increased viability from ~72% (parental J82-Ras) to 90% but still susceptible to Rom+Cis+Gem (Fig. 5h). Our in vivo study showed that GC-resistant J82-Ras CDXs still responded to the Gem plus Rom+Cis regimen but not Gem plus Cis regimen (Fig. 5i). Analysis of final tumour volume revealed a growth of CDXs for 17 days to ~395%, ~360%, and ~211% of their original volume in mice treated with PBS, Gem plus Cis, and Gem plus Rom+Cis, respectively (Fig. 5j). Comparing tumour weights showed that final T/C of tumours isolated at day 17 from mice treated with Gem plus Cis, and Gem plus Rom+Cis were at 0.81/1.0 and 0.44/1.0, respectively. The results verified the ability of Gem plus Rom+Cis to control GC-resistant CDXs.

### Efficacy of Gem plus Rom+Cis in controlling T24 CDX

**Fig. 6:**
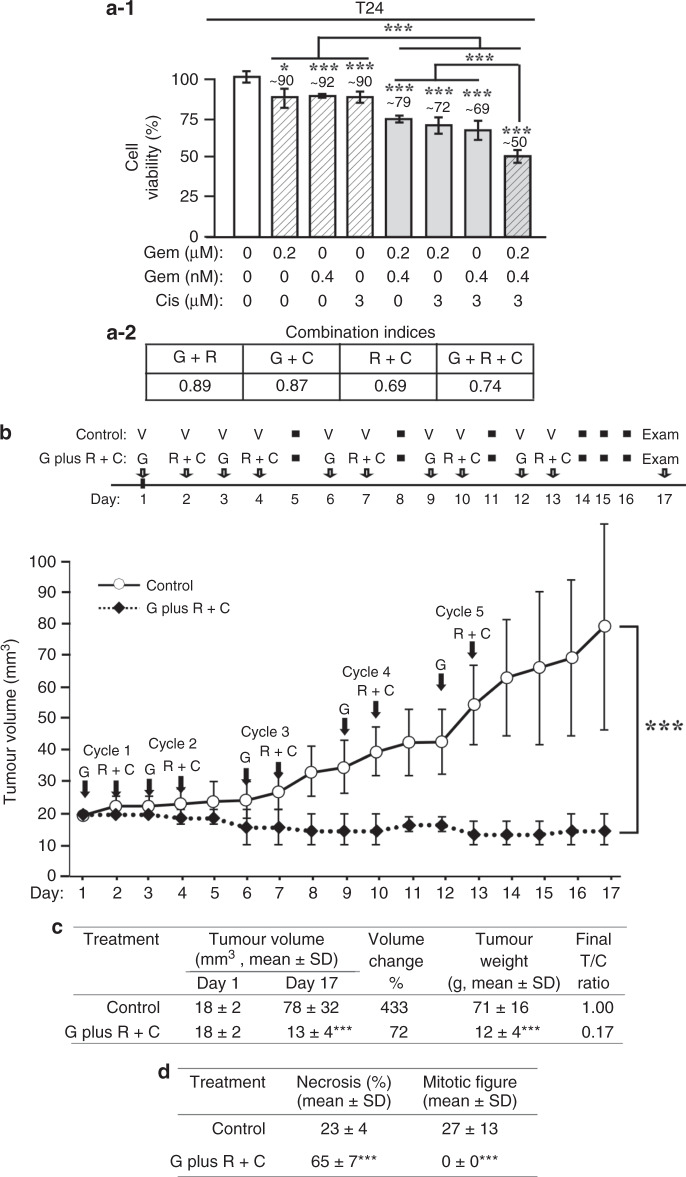
Efficacy of Gem plus Rom+Cis in controlling T24 CDX. **a-1**, **a-2** T24 cells were treated with Gem (G), Rom (R), and/or Cis (C) for 48 h, cell viability was determined (**a-1**), and combined effects were calculated (**a-2**) to reveal combination indices <1 for synergistic effects. **b** In all, 5 × 10^6^ T24 cells were mixed with Matrigel and inoculated into the flank areas of nude mice. Tumour volume was measured with a calliper and determined with a formula (length × width^2^ × ½).^43^ Mice developing CDX tumours reaching ~18 mm^3^ were entered into the treatment study (day 1). Nude mice, four per group, were administered (i.p.) PBS (V, control) and Gem plus Rom+Cis (G plus R+C) at the indicated days for five treatment cycles. Tumour volume was measured daily. Mice were histopathologically examined during necropsy at day 17. **c** Tumour volume at days 1 and 17 is presented as mean ± SD. Changes in tumour volume (%) were calculated by T17 (tumour volume determined at day 17)/T1 (tumour volume determined at day 1). Average weight of tumours isolated at day 17 was measured as mean ± SD. Final tumour/control ratio (T/C) was calculated by T (mean tumour weight of treatment group)/C (mean tumour weight of control group) of tumours isolated from mice at day 17. **d** Tumours isolated from mice treated with PBS or Gem plus Rom+Cis were histologically examined to determine necrosis and mitotic figure. Necrosis (%) and mitotic cells were determined and averaged from 10 HPFs, presented as mean ± SD. Statistical significance is indicated by *p < 0.05, ***p < 0.001.

To determine the ability of the Gem plus Rom+Cis regimen to control CDXs was not limited to J82-Ras CDXs, we included the tumorigenic UC T24 cell line, which carries the endogenous oncogenic *H-Ras* gene. Initially, we treated T24 cells with Gem, Rom, and/or Cis at their IC_10_ doses and detected that double and triple combinations of these agents synergistically induced cell death, and Rom+Cis+Gem was more effective than double combinations in inducing cell death (Fig. 6a-1, a-2). Subsequently, we determined that Gem plus Rom+Cis was also highly efficacious in controlling T24 CDX development (Fig. 6b). Analysis of final tumour volume revealed the growth of CDXs in 17 days to ~433% and a reduction to ~72% of their original volume in mice treated with PBS and Gem plus Rom+Cis, respectively (Fig. 6c). Comparing tumour weights (final T/C at 0.17/1.00) verified the ability of Gem plus Rom+Cis to effectively control T24 CDXs. Histological examination revealed an average of ~27 and 0 mitotic cells and ~23% vs. ~65% necrosis area per HPF in tumour tissues isolated from mice treated with PBS and Gem plus Rom+Cis, respectively (Fig. 6d). These results indicated that the Gem plus Rom+Cis regimen was also effective in inhibiting proliferation and inducing death of T24 CDX tumour cells in vivo, and the ability of Gem plus Rom+Cis to control CDXs was not limited to one UC cell type.

## Discussion

In this communication, we demonstrated, for the first time, that the triple combination Gem plus Rom+Cis regimen was highly efficacious and more efficacious than the standard double combination Gem plus Cis regimen in suppressing cell viability, drug resistance, and CDX development of the UC J82, J82-Ras, T24, and/or SW780 cells. Gem plus Rom+Cis was also efficacious in controlling GC-resistant J82-Ras CDXs. Our results strongly suggest that the Gem plus Rom+Cis regimen should be seriously considered to control UC malignancy and recurrence.

Our in vitro studies indicated that Gem+Rom+Cis was able to synergistically induce apoptotic cell death and reduce drug resistance/clonogenic resistance of UC cells more effectively than double combinations. Gem+Rom+Cis also showed a preferential suppression of viability and drug resistance in the oncogenic H-Ras-expressing J82-Ras vs. J82 cells. Our transcriptome analysis revealed that a higher level of cell death-associated genes was significantly induced by Gem+Rom+Cis in J82-Ras than in J82 cells, and ROS-associated genes were significantly induced in J82-Ras but not in J82 cells. In contrast, drug resistance-associated genes were significantly induced in J82 but not in J82-Ras cells. In addition, higher numbers of DNA repair-, autophagy-, and drug transport-associated genes were induced by Rom+Cis+Gem in J82 than in J82-Ras cells. These discrepancies support the mechanism for Gem+Rom+Cis to preferentially induce cell death and reduce drug resistance in J82-Ras cells vs. J82 cells. More than 80% of UCs are associated with the aberrant induction of the growth factor receptor (GFR) to the Ras-ERK pathway.^44,45^ Our in vivo studies verified that the Gem plus Rom+Cis regimen was highly efficacious in controlling CDXs of J82-Ras and T24, which carries the oncogenic endogenous H-*Ras* gene. Thus the Gem plus Rom+Cis regimen may particularly target advanced UCs with aberrant GFR-Ras-ERK pathways.

Induction, instead of suppression, of the ERK-Nox pathway and ROS played important roles in Gem+Rom+Cis-induced apoptosis. The Ras-ERK pathway is often associated with cell proliferation and survival of cancers.^46^ Mek inhibitors, such as binimetinib and trametinib, have been shown to suppress tumours, such as melanomas, where the ERK pathway is overactive.^46–48^ However, using Mek inhibitors alone often comes with drug resistance due to the feedback reactivation of the ERK pathway.^46,48^ Thus Mek inhibitors are used in combination with other drugs, such as binimetinib combined with the B-Raf inhibitor encorafenib, to treat cancers and prevent drug resistance.^47,48^ In contrast, our studies indicate that enhancing the ERK pathway by anticancer regimens, containing romidepsin, instead of suppressing the ERK pathway, results in activating the ERK pathway to induce Nox1 and elevate ROS, leading to enhanced cell death in various cancer cells, including urinary bladder, breast, and colorectal cancer cells.^18–21,49^ In these studies, using the Mek inhibitor U0126 to block the ERK pathway to the downstream Nox-ROS pathway attenuated romidepsin-induced cell death, clearly indicating that the romidepsin-induced ERK pathway is essential for cell death but not for survival. Our studies revealed that ROS elevation was required for inducing the ERK-Nox pathway, and the ERK-Nox pathway was also required for ROS elevation. Gem and Cis have been shown to induce mitochondrial ROS for inducing cell death.^31,50^ Nox family members play major roles in ROS production.^29,33^ Accordingly, Gem+Rom+Cis may induce both mitochondria- and Nox-dependent ROS to jointly elevate ROS to a lethal level, causing caspase activation, PARP proteolysis, DNA oxidation, and DNA damages. In addition, Gem inhibits DNA replication,^51^ and Cis damages DNA.^35^ It is conceivable that Gem+Rom+Cis induced DNA damage and inhibited DNA repair directly, induced ROS-dependent DNA oxidation and damage, and inhibited PARP-dependent DNA repair, thereby holistically contributing to synergistic induction of cell death and suppression of clonogenic survival/drug resistance. We detected that Rom+Cis induced higher levels of cell death than Cis+Gem, but Cis+Gem was more effective than Rom+Cis in suppressing clonogenic survival. Apparently, mechanisms for inducing cell death were not fully overlapped with suppressing clonogenic survival. We also detected that Cis was less effective than Rom and Gem in reducing GSH; however, Cis facilitated Rom and Gem to reduce GSH and clonogenic survival. GSH is the most abundant intracellular antioxidant and forms conjugates with Cis for exportation, resulting in resistance to Cis.^3,35,52,53^ Thus a combination of Gem+Rom+Cis was complementary to become more effective than any double combinations in inducing cell death and reducing clonogenic survival/drug resistance.

Our investigation revealed the key UPR modulator BiP induced by Gem+Rom+Cis to support cell death. However, others showed the role BiP plays in supporting tumorigenesis, anti-apoptosis, and drug resistance to Gem.^8,39,40^ Our gain- and loss-of-function studies indicated that ectopic expression, but not knockdown, of BiP resulted in increased susceptibility of cells to Gem+Rom+Cis for death, indicating a novel role of elevated BiP played in supporting apoptosis, but not anti-apoptosis, in response to Gem+Rom+Cis. Although BiP has been reportedly associated with poor prognosis and chemo-resistance in pancreatic, brain, liver, lung, and breast cancers,^54^ BiP has also been shown to play a role in inducing apoptosis.^55–57^ BiP has been shown to interact with the secreted protein acidic and rich in cysteine (SPARC) to induce apoptosis through the PERK/eIF2α and IRE1α/XBP1 pathways in colorectal cancer cells.^55^ BiP interacts with the prostate apoptosis response-4 (Par-4) at the cell surface to activate FADD/caspase-8/caspase-3 pathway and induce extrinsic apoptosis.^55^ Par-4 is a tumour suppressor that is usually downregulated in oncogenic Ras-expressing cells,^58,59^ and overexpression of Par-4 enhances apoptosis in oncogenic Ras-expressing fibroblasts.^58^ Romidepsin treatment may result in elevating Par-4 level, and overexpression of Par-4 sensitises recurrent tumours to chemotherapy.^59^ Cisplatin treatment may enhance the apoptosis of Par-4-expressing Wilms’ tumour cells through the endoplasmic reticulum apoptotic pathway with Par-4 interaction with BiP.^57^ However, the extent to which Par-4 and/or the SPARC to PERK/eIF2α and IRE1α/XBP1 pathways are involved in BiP-mediated cell death induced by Gem+Rom+Cis remains to be clarified.

The current standard regimen for advanced UCs is Gem plus Cis.^5,60,61^ Our studies demonstrated that integration of Rom into the double combination regimen, resulting in the Gem plus Rom+Cis regimen, may significantly improve its efficacy in controlling UCs. We formulated the dose-reduced combination Gem plus Rom+Cis regimen with less than 6% Gem, 25% Rom, and 25% Cis of their clinically equivalent doses that was well tolerated in animals. Because Gem, Rom, and Cis are FDA-approved to treat cancers, the safe dose-reduced Gem plus Rom+Cis regimen shall be rapidly translated into clinical studies to improve chemotherapy for controlling the development and recurrence of advanced UCs, especially Ras-ERK-activated UCs, ultimately improving patients’ quality of life.

## Supplementary information

Combined supplementary materials

## Data Availability

All the data related to this study are included in this article and its supplementary file.
