# Supplementary material for: Compensatory combination of romidepsin with gemcitabine and cisplatin to effectively and safely control urothelial carcinoma
Source: Br J Cancer. 2020 May 11;123(2):226–39. doi: 10.1038/s41416-020-0877-8 (PMC7374627; doi:10.1038/s41416-020-0877-8)

Supplementary Tables

Supplementary Table S1: Concentrations of agents used in vitro studies.

Cell line	Gemcitabine (μM)	Romidepsin (nM)	Cisplatin (μM)	NAC (mM)	U0126 (μM)	ML171 (μM)
J82	0.4	1	3	5	2.5	1
J82-Ras	0.2	0.5	3	5	2.5	1
SW780	0.4	0.7	10	5	2.5	1
T24	0.2	0.4	3	5	2.5	1

Supplementary Table S2: Treatment dosage and schedule for in vivo studies.

Regimen	Drug dosage	Schedule
Gem plus Cis	20 mg/kg of Gem in PBS, IP<br>5 mg/kg of Cis in PBS, IP	Day 1, 3, 6, 9 & 12<br>Day 2, 4, 7, 10 & 13
Rom +Cis	1 mg/kg of Rom+ 5 mg/kg of Cis in PBS, IP	Day 1, 3, 6, 9 & 12
Gem plus Rom+Cis	20 mg/kg of Gem in PBS, IP<br>1 mg/kg of Rom+ 5 mg/kg of Cis in PBS, IP	Day 1, 3, 6, 9 & 12<br>Day 2, 4, 7, 10 & 13

Gem: Gemcitabine**; Rom:** Romidepsin**; Cis:** Cisplatin

Supplementary Table S3: Specific antibodies used in immunoblotting.

Antibody	Company	Catalogue number	Dilution	Blocking solution
H-RAS	SCB	SC-520	1:500	5% NDM
MOX1 (Nox-1)	SCB	SC-518023	1:500	5% NDM
p-MEK kinase-1	SCB	SC-13020	1:1000	5% BSA
MEK kinase-1	SCB	SC-49449	1:1000	5% NDM
p-ERK	SCB	SC-7383	1:500	5% BSA
ERK-2	SCB	SC-154	1:1000	5% NDM
Actin	SCB	SC-1616	1:500	5% NDM
PARP	CST	9542S	1:1000	5% NDM
BiP	CST	3177S	1:1000	5% NDM

SCB: Santa Cruz Biotechnology; **CST:** Cell Signaling Technology, **NDM**: Nonfat dried milk; **BSA**: Bovine serum albumin

Supplementary Table S4: Standard protocols of clinical anti-cancer regimens converted for mouse use.

Regimen	Cancer type	Drug dosage in humans	Drug dosage converted for mouse study	Schedule for humans
Gem plus Cis	UC	Gem, 1000 mg/m^2<br>Cis, 70 mg/m^2	Gem, 324 mg/kg<br>Cis, 23 mg/kg	Day 1, 8 & 15<br>Day 2
Rom	Lymphoma and Refractory solid tumor	Rom, 8-17.5 mg/m^2	Rom, 5 mg/kg	Day 1, 8 & 15

UC: Urothelial carcinoma; **Gem:** gemcitabine; **Rom:** romidepsin; **Cis:** cisplatin

Supplementary Figure Legends

Supplementary Figure S1. Densitometric analysis of Mek, Erk, and Nox levels. As performed in **Fig. 1g**, J82 and J82-Ras cells were treated with Rom, Cis, and/or Gem at their IC_{10} doses for 24 h. Cell lysates were prepared and analyzed by immunoblotting using specific antibodies to detect levels of phosphorylated Mek1/2 (p-Mek1/2), Mek1/2, phosphorylated Erk1/2 (p-Erk1/2), Erk1/2, and Nox-1, with β-actin as a control, and these levels were quantified by densitometry. Levels of specific phosphorylation of Mek1/2 (**a**, p-Mek/Mek) and Erk1/2 (**b**, p-Erk/Erk) were calculated by normalizing the levels of p-Mek1/2 and p-Erk1/2 with the levels of Mek1/2 and Erk1/2, respectively, then the level was set in untreated control cells as 1 (X, arbitrary unit). Levels of Nox-1 (**c**, Nox-1/actin) were calculated by normalizing with the level of β-actin and the level set in control cells as 1 (X, arbitrary unit). Columns, mean of triplicates; bars, SD. The *p* value was adjusted for multiple comparisons by using the Simes method. Statistical significance, indicated by $^{*}p < 0.05$, $^{**}p < 0.01$, $^{***}p < 0.001$. All results are representative of three independent experiments.

Supplementary Figure S2. H-Ras and Erk in J82 and J82-Ras cells. (**a**) Cell lysates of J82 and J82-Ras cells were prepared and analyzed by immunoblotting using specific antibodies to detect levels of H-Ras, with β-actin as a control. Levels of H-Ras (H-Ras/actin) were calculated by normalizing with the level of β-actin and the level set in control cells as 1 (X, arbitrary unit). (**b**) J82 and J82-Ras cells were treated with Gem+Rom+Cis (G+R+C) for 24 h. Cell lysates were prepared and analyzed by immunoblotting using specific antibodies to detect levels of phosphorylated phosphorylated Erk1/2 (p-Erk1/2) and Erk1/2, with β-actin as a control. These

levels were quantified by densitometry. Levels of specific phosphorylation of Erk (p/Erk) were calculated by normalizing the levels of p-Erk1/2 with the levels of Erk1/2, respectively, then the level was set in untreated control cells as 1 (X, arbitrary unit). All results are representative of three independent experiments.

Supplementary Figure S3. Rom+Cis+Gem synergistically induced death and suppressed drug resistance in SW780 cells. SW780 cells were treated with Rom, Cis, and/or Gem at their IC_{10} doses in the absence or presence of NAC, U0126, or ML171. (**a-1**) Cell viability was determined, and relative cell viability was normalized by the value determined in untreated counterpart cells, set as 100%. (**a-2**) Combined effects (**a-1**) were determined to reveal combination indices <1 for synergistic effects. (**b**) Cell lysates were prepared and analyzed by immunoblotting using specific antibodies to detect levels of p-Erk1/2, Erk1/2, and Nox-1, with β-actin as a control, and these levels were quantified by densitometry. Levels of specific phosphorylation of Erk1/2 (p/Erk) were calculated by normalizing the level of p-Erk1/2 with the level of Erk1/2, then the level set in control cells as 1 (X, arbitrary unit). Levels of Nox-1 (Nox/actin) were calculated by normalizing with the level of β-actin and the level set in control cells as 1 (X). (**c & g**) Relative ROS levels were measured and normalized by the level determined in untreated counterpart cells, set as 1 (X). (**d**) Relative caspase-3/7 activity was determined and normalized by cell viability, and then the relative values were normalized by the value determined in untreated counterpart cells, set as 1 (X, arbitrary unit). (**e**) DNA oxidation was measured by an Fpg-modified comet assay and normalized by the value of average tail moment determined in untreated control cells, set as 1 (X). (**f**) DNA damage was measured by an alkaline comet assay and normalized by the value of average tail moment determined in

untreated control cells, set as 1 (X). (**h**) Apoptotic cell population (%) was measured by flow cytometry with an annexin-V-FITC apoptosis detection kit. (**i**) Clonogenic survival was measured by a clonogenic assay. Relative colony formation was normalized by the value determined in untreated counterpart cells, set as 100%. (**j**) GSH content was determined and relative GSH level was normalized by the value determined in untreated counterpart cells, set as 100%. Columns, mean of triplicates; bars, SD. The *p* value was adjusted for multiple comparisons by using the Simes method. Statistical significance is indicated *$p < 0.05$, **$p < 0.01$, ***$p < 0.001$. All results are representative of three independent experiments.

Supplementary Figure S4. BiP induced by Rom+Cis+Gem in T24 and SW780cells. (**a**) T24 and (**b**) SW780 cells were treated with Gem+Rom+Cis (G+R+C) for 48 h. Cell lysates were prepared and analyzed by immunoblotting using specific antibodies to detect levels of BiP, with β-actin as a control, and these levels were quantified by densitometry. Levels of BiP (BiP/actin) were calculated by normalizing with the level of β-actin and the level set in control cells as 1 (X, arbitrary unit). All results are representative of three independent experiments.

Supplementary Figure S5. TUNEL assay. (**a**) J82-Ras cultures were treated with Rom+Cis+Gem for 24 h, rinsed, and fixed with 3.7% formaldehyde. (**b**) Paraffin embedded tissues were deparaffinized and rehydrated. Then, cultures and tissues were examined with the TACS 2 TdT-DAB In Situ Apoptosis Detection Kit (Trevigen, MD, USA) to detect apoptotic cells. Samples were counter-stained by methyl green. Images were taken at 400x; scale bar, 50 μm. Black arrows indicate apoptotic cells.

Supplementary Figure S1

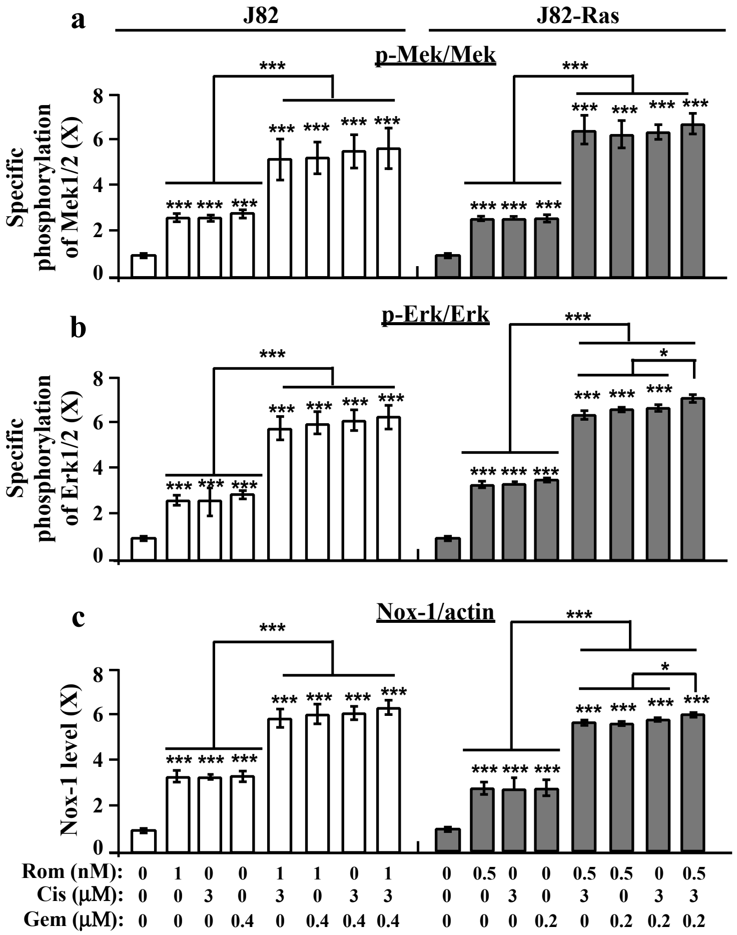

Supplementary Figure S2

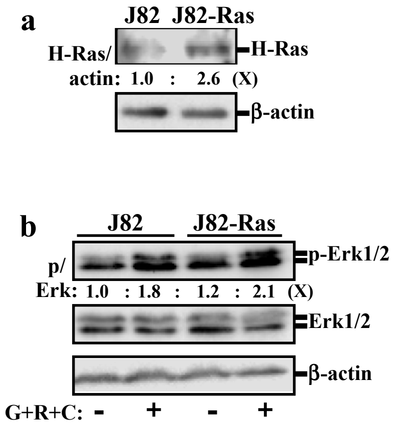

Supplementary Figure S3

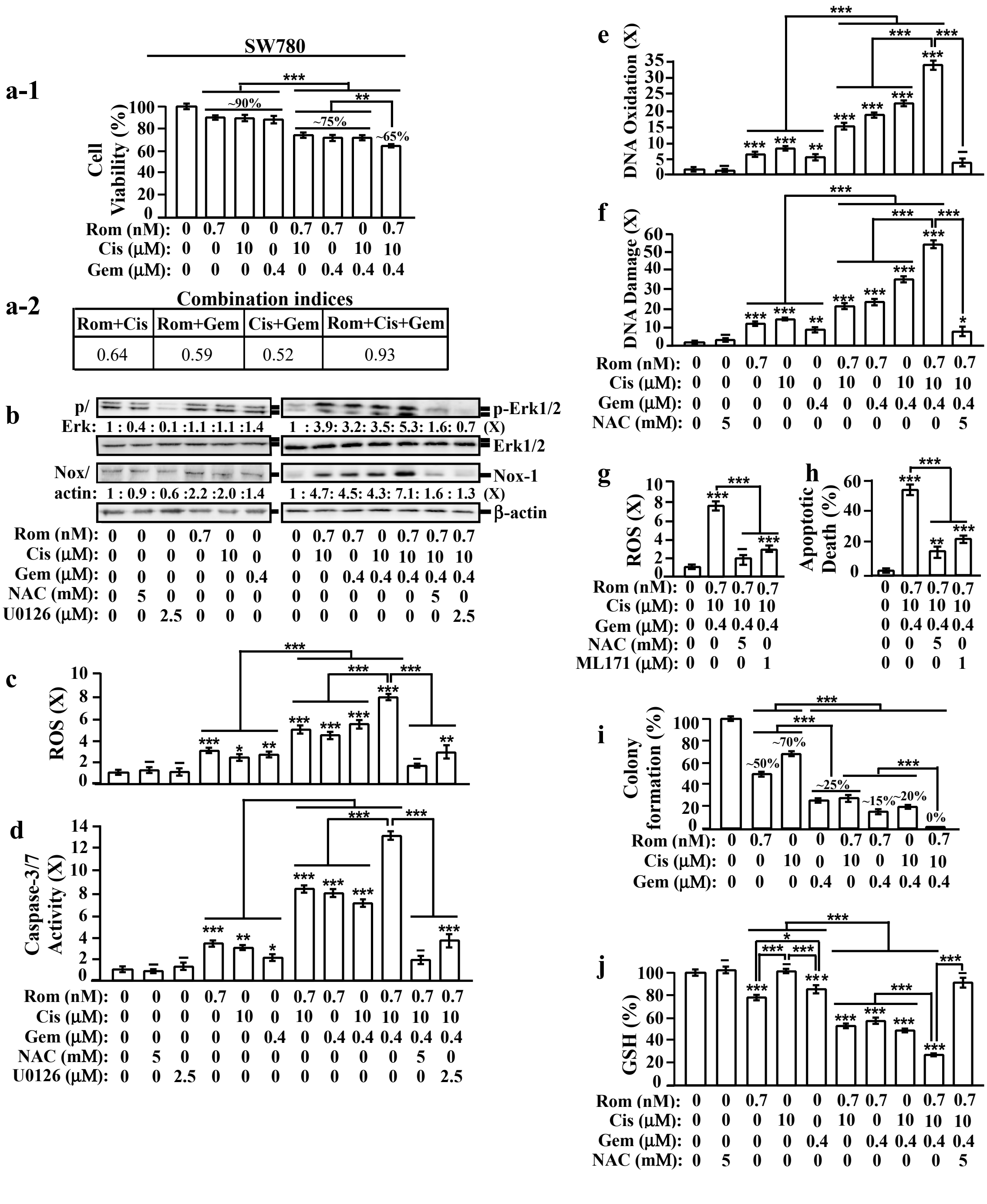

Supplementary Figure S4

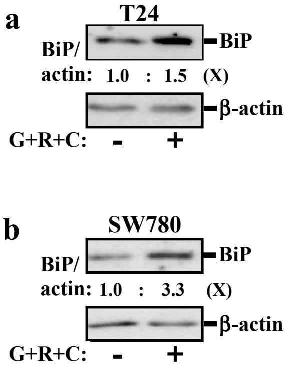

Supplementary Figure S5

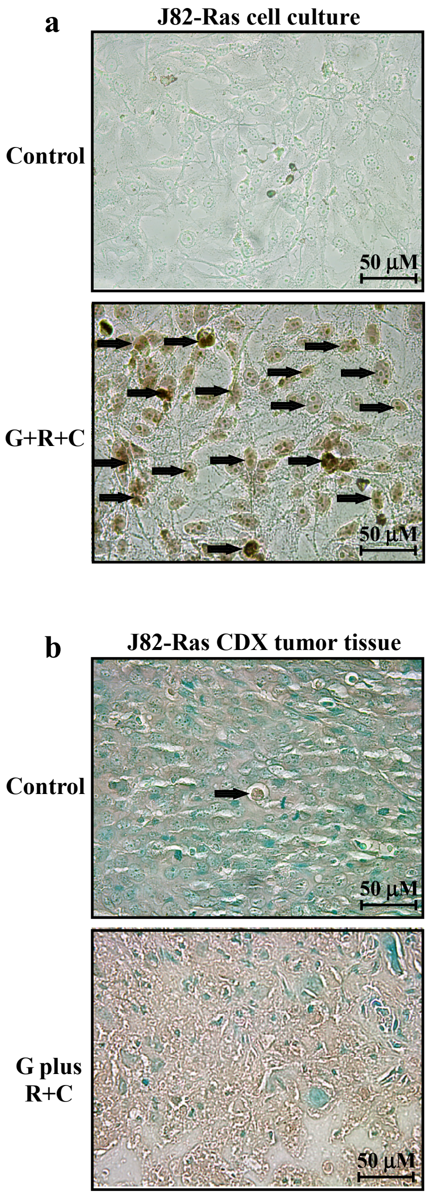

Supplement: Supplementary file 1 — Combined supplementary materials [file 41416_2020_877_MOESM1_ESM.pdf]
